# Preparation of a Herbal Mouthwash With Lemongrass and Mint-Mediated Zinc Oxide Nanoparticles and Evaluation of Its Antimicrobial and Cytotoxic Properties

**DOI:** 10.7759/cureus.53671

**Published:** 2024-02-05

**Authors:** Rajeshkumar Shanmugam, Sulochana Govindharaj, Padmapriya Arunkumar, Ganji Sai Sanjana, Pradeep Manigandan

**Affiliations:** 1 Nanobiomedicine Lab, Centre for Global Health Research, Saveetha Medical College and Hospital, Saveetha Institute of Medical and Technical Sciences, Saveetha University, Chennai, IND; 2 Pharmacology, Saveetha Dental College and Hospitals, Saveetha Institute of Medical and Technical Sciences, Saveetha University, Chennai, IND

**Keywords:** zinc oxide nanoparticles, herbal mouthwash, cytotoxic effect, antimicrobial activity, product development, eco-friendly

## Abstract

Introduction

Nanotechnology holds considerable importance in biomedical and dental applications. Nanoparticles synthesized using green synthesis methods with herbal formulations offer various benefits to humans. Zinc oxide nanoparticles (ZnONPs), being semiconductors, exhibit potent antibacterial properties. Notably, treatments utilizing lemongrass and mint ensure potentially lower toxicity and antibacterial qualities for oral infections. The goal of the study is to prepare a mouthwash mediated by ZnONPs and assess its cytotoxic potential and antibacterial efficacy.

Materials and methods

A lemongrass and mint formulation was used in the synthesis of ZnONPs, and the mouthwash was prepared using the synthesized nanoparticles. The produced ZnONPs were tested for their antimicrobial activity using agar well diffusion technique against oral pathogens, and the ZnONPs-mediated mouthwash was evaluated for its cytotoxic effect using the brine shrimp lethality assay and compared to commercial mouthwash.

Results

The green-synthesized ZnONPs were initially confirmed using a UV-visible spectrophotometer and exhibited a maximum peak at 362 nm. The antimicrobial activity was tested for the synthesized ZnONPs against oral pathogens, which showed a maximum zone of inhibition of 22 mm in *Enterococcus faecalis* and 23 mm in *Candida albicans*, as estimated by the agar well diffusion technique. Additionally, ZnONPs-based herbal mouthwash demonstrated lower cytotoxicity than the commercial mouthwash in the brine shrimp lethality assay.

Conclusion

In the current study, lemongrass and mint-mediated ZnONPs demonstrated an effective antibacterial activity against *E. faecalis* and antifungal activity against* C. albicans*. Furthermore, the cytotoxic effect tested using the brine shrimp lethality assay for ZnONPs-mediated mouthwash demonstrated lower toxicity as compared to the commercial mouthwash. This suggests that the green-synthesized ZnONPs-based mouthwash could be used as an alternative to synthetic mouthwash.

## Introduction

Nanotechnology is an emerging technology with multiple applications in the medicinal, chemical, cosmetics, and food processing industries. It plays a vital role in drug delivery, diagnostic techniques, antibacterial treatments, wound treatment, and managing diseases like cancer, heart disease, diabetes, and kidney diseases [1]. The incorporation of various nanoparticles that are green-synthesized from plants is an emerging method with as yet unknown medicinal benefits. The enhancement of medical implementations of nanobiotechnology has ushered in a new era of nanomedicine by introducing the idea of controlling and treating the biological systems of humans using nanomaterials [2].

The unique features of metal oxide nanoparticles, such as their optical properties, superparamagnetic behavior, selective catalytic activity, and sensitivity, have attracted huge attention among researchers. Since zinc oxide nanoparticles (ZnONPs) are generally recognized as safe (GRAS) and less toxic, they are frequently chosen over other metal oxide nanoparticles [3]. ZnONPs possess several biomedical applications such as antibacterial activity, anti-inflammatory activity, antidiabetic activity, and anti-cancer effect. They are also employed in wastewater treatment to remove impurities, heavy metals, and organic pollutants by photodegradation [4]. ZnONPs are well-known for their efficient antibacterial activity against various bacterial strains [5]. Due to the decreased size of ZnONPs, they show potential antibacterial effects because they can efficiently enter the cell and eradicate the microorganisms. ZnONPs are also used in dental applications in the development of value-based products such as toothpaste, mouth rinse, dental varnish, and mouthwash [6]. Chemical and green synthesis of nanoparticles are the two common methods used for the synthesis of nanoparticles, where the green synthesis method is eco-friendly, low-cost, and effective. In addition, various plants, seaweed, and marine microalgae contain diverse bioactive compounds such as polysaccharides, pigments, and phenolic compounds that can act as reducing and stabilizing agents used in the synthesis of ZnONPs.

Lemongrass (*Cymbopogon citratus*), derived from the Cymbopogon family, contains a significant amount of bioactive compounds, such as citral (mixture of geranial and neral), isoneral, isogeranial, geraniol, geranyl acetate, citronellal, citronellol, germacrene-D, and elemol, and exhibits numerous promising activities such as anti-inflammation, antibacterial properties, anti-cancer effects, pain relief, and alleviation of common allergies related to dental applications [7]. The therapeutic efficacy of herbal-based formulations is associated with secondary metabolites commonly present in plants. Mint (*Mentha*), from the Lamiaceae family, contains menthol, an organic compound naturally found in peppermint and other mint plants [8,9]. Secondary metabolite compounds such as p-coumaric acid, furfural, phenolic compounds, anthocyanin, phytosterols, organic acids, methyl heptenone, and isopulegol are present in mint [10,11]. This study proposes employing a green synthesis method to produce ZnONPs by using a formulation of lemongrass and mint extracts, which is cost-effective and eco-friendly, has lower energy consumption, and requires very minimum technical expertise and equipment.

The current study involves the green synthesis of ZnONPs with lemongrass and mint formulation. To evaluate their antimicrobial activity, an agar well diffusion technique was utilized to test against oral pathogens compared to standard. Furthermore, we used the brine shrimp lethality test to evaluate the cytotoxicity effect of the prepared ZnONPs-based herbal mouthwash compared to commercial mouthwash.

## Materials and methods

Extraction of herbal formulation

​Lemongrass leaves and mint leaf powder were collected from a commercial store. One gram of lemongrass powder and one gram of mint leaf powder were added to 100 mL of distilled water [12]. The mixture was heated in a mantle for 15-20 minutes at 40-50°C. After heating, the solution was cooled and filtered using Whatman No. 1 filter paper (Whatman Plc, Maidstone, UK). The resulting formulation was then used for further studies [13].

Preparation of ZnONPs

A total of 30 mM of zinc nitrate was dissolved in 50 mL of distilled water to make a zinc nitrate solution. Subsequently, 50 mL of the lemongrass and mint formulation was added to the prepared zinc nitrate solution. The resulting mixture solution was placed in a magnetic stirrer set at 600-700 rpm (rotations per minute). Nanoparticle synthesis was analyzed using a UV-visible double-beam spectrophotometer. After 24 hours, the solution was removed from the stirrer to record readings and observe color changes. The solution gradually darkened over time compared to its initial color. The nanoparticles were then centrifuged at 8,000 rpm for 10 minutes, and the pellet was collected and kept in a hot air oven to convert it into powder. From that, 100 mg of the nanoparticles were dissolved in 10 mL of distilled water for further processing.

Preparation of mouthwash

The mouthwash was prepared using 0.3 g of sucrose as a sweetener, 0.001 g of sodium benzoate as a preservative, and 0.01 g of sodium lauryl sulfate as a foaming agent. These were dissolved in 9.5 mL of distilled water. Subsequently, 500 μg/mL of lemongrass and mint-mediated ZnONPs were added to the mouthwash preparation, and the mixture was placed in an orbital shaker. Finally, a total volume of 10 mL of lemongrass and mint-formulated ZnONPs-based mouthwash was used for further studies [14].

Antimicrobial activity

The antimicrobial test for lemongrass and mint formulation-mediated ZnONPs was conducted using an agar well diffusion method. For the antimicrobial assay, the Muller Hinton agar was employed for bacterial culture while the Rose Bengal agar was used for fungal culture. Isolates of *Staphylococcus aureus, Streptococcus mutans, Enterococcus faecalis, *and* Candida albicans *were spread using sterile cotton swabs. A 9 mm diameter well was created in each agar plate using a sterile polystyrene tip. The wells were then filled with various concentrations of lemongrass and mint-mediated ZnONPs (25 μg/mL, 50 μg/mL, 100 μg/mL). Amoxyrite was used as a standard for bacteria and fluconazole for fungi at a concentration of 25 μg/mL. The petri plates were incubated for 24 hours for bacterial cultures and 48 hours for fungi cultures at 37 degrees Celsius. The antimicrobial activity was assessed by measuring the diameter of the zone of inhibition around the respected wells [15].

Cytotoxic effect using brine shrimp lethality assay

In 200 mL of distilled water, 2 g of iodine-free salt was added and mixed thoroughly. A six-well ELISA (enzyme-linked immunosorbent assay) plate reader was taken, and 10-12 mL of saline water was added carefully. Subsequently, 10 nauplii were slowly added to each well. The prepared ZnONPs-based mouthwash was then added to each well at different concentrations (5 μg/mL, 10 μg/mL, 20 μg/mL, 40 μg/mL, 80 μg/mL). The sixth well was used as a control (containing only salt water and live nauplii, without any samples) as noted in previous studies [16]. A comparison was made between the ZnONPs-based mouthwash and commercial mouthwash. The ELISA plate was left for incubation for approximately 24 hours. After incubation, the plates were analyzed and the number of live nauplii present was noted down. The percentage of live nauplii was estimated using the following formula:

(Number of dead nauplii/Number of dead nauplii + Number of live nauplii) × 100

## Results

Preliminary characterization of green-synthesized ZnONPs

**Figure 1:**
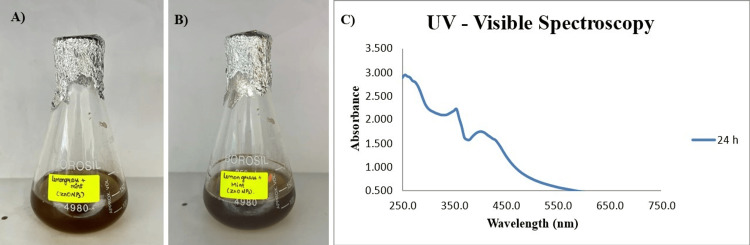
Synthesized ZnONPs and UV-visible spectroscopy readings (A) Initial color of ZnONPs solution; (B) Final color change of ZnONPs solution; (C) UV-visible spectrum of the green-synthesized ZnONPs with a peak at 362 nm, confirming the presence of synthesized nanoparticles. ZnONPs: Zinc oxide nanoparticles

After incubation for 24 hours, a color change from light to darkish brown was observed, indicating the lemongrass and mint formulation's reducing and capping ability. The color reduction is represented in Figure 1 (A, B). The absorbance of the prepared ZnONPs was measured using a UV-visible spectrophotometer, and the highest peak was observed around 362 nm, displaying the synthesis of ZnONPs. Figure 1 (C) shows the graphical representation of the lemongrass and mint-formulated ZnONPs' absorbance from 250 to 650 nm in a UV-visible spectrophotometer.

Antimicrobial activity

**Figure 2:**
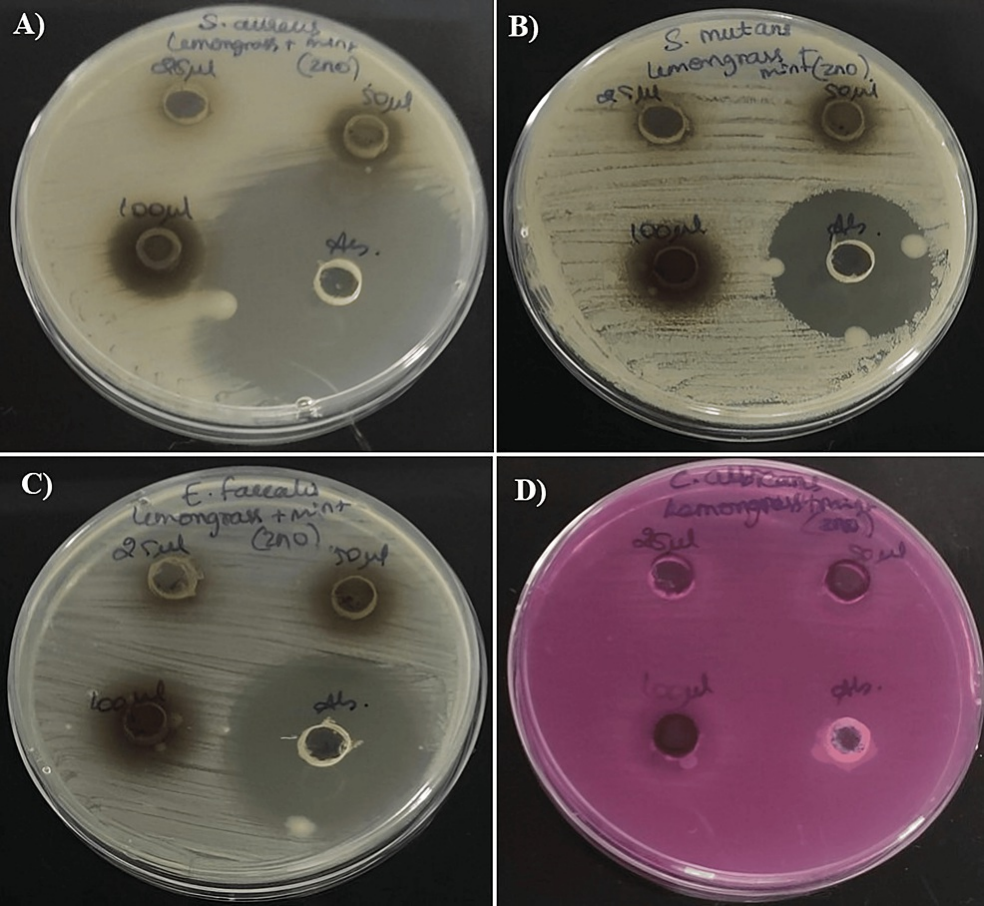
Antimicrobial activity of lemongrass and mint-mediated ZnONPs against oral pathogens (A) Staphylococcus​​​​​​​ aureus, (B) Streptococcus mutans, (C) Enterococcus faecalis, and (D) Candida albicans ZnONPs: Zinc oxide nanoparticles

**Figure 3:**
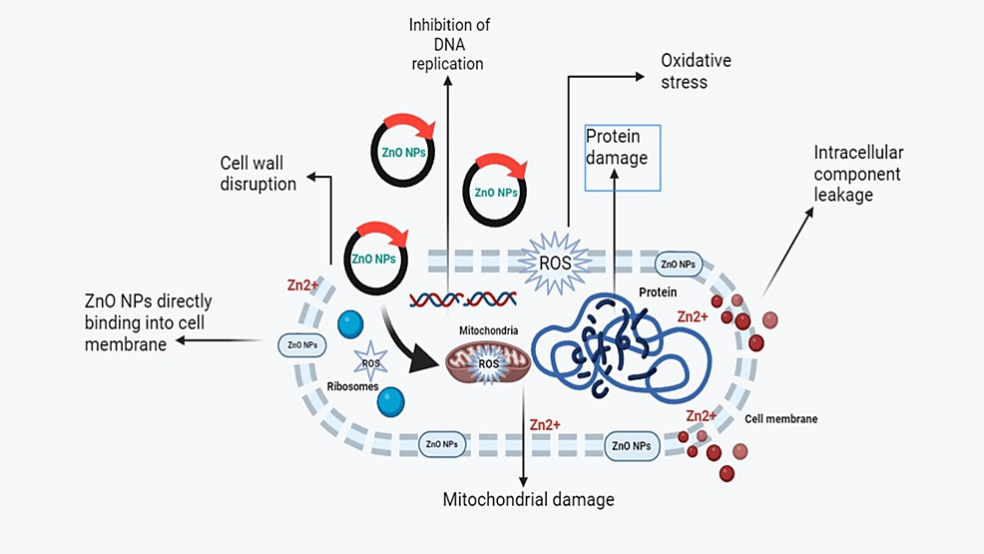
Diagrammatic illustration of antibacterial activity of ZnONPs ZnONPs: Zinc oxide nanoparticles; ROS: Reactive oxygen species; Zn^2+^: Zinc ions

Lemongrass and mint-based ZnONPs were tested against four oral pathogens: A) *S. aureus*, B) *S. mutans*, C) *E. faecalis*, and D) *C. albicans*. For the standard, amoxicillin was used for bacteria and fluconazole for fungi. At the highest concentration of 100 μg/mL, the maximum zone of inhibition was observed against the tested organisms: *E. faecalis* (21 mm), *S. aureus* (20 mm), *S. mutans* (19 mm), and *C. albicans* (20mm). In the case of *C. albicans*, the prepared nanoparticles exhibited potent antimicrobial activity compared to the standard, which showed no inhibition. These findings suggest that lemongrass and mint-based ZnONPs have strong potential as antimicrobial agents, as depicted in Figure 2. A diagrammatic illustration of the antibacterial mechanism of ZnONPs is presented in Figure 3.

Cytotoxic effect using brine shrimp lethality assay

**Figure 4:**
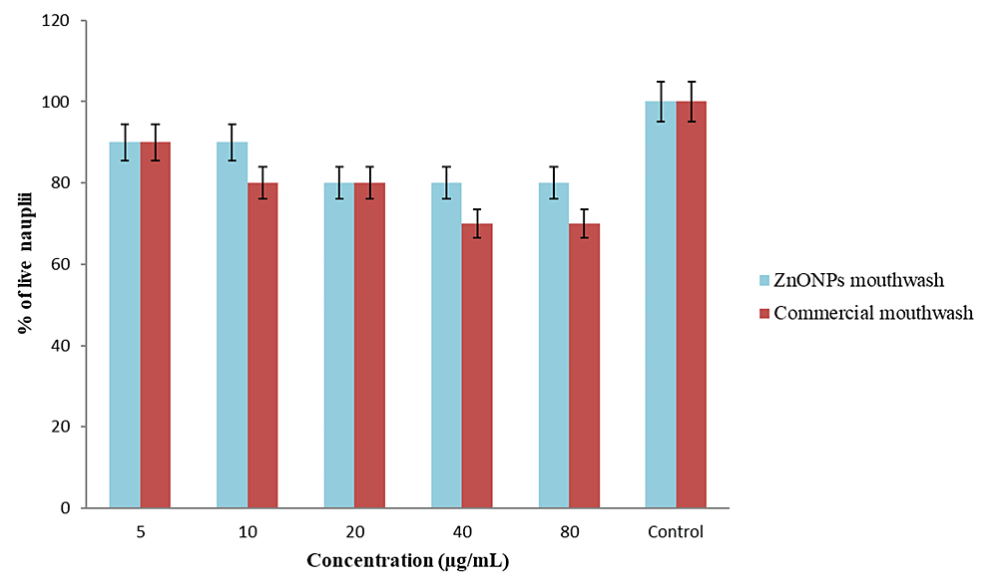
Cytotoxic effect of lemongrass and mint-mediated ZnONPs-based mouthwash using brine shrimp lethality assay ZnONPs: Zinc oxide nanoparticles

In Figure 4, the cytotoxic effect of the mouthwash prepared with lemongrass and mint formulation-mediated ZnONPs was compared to that of commercial mouthwash, using different concentrations ranging from 5 μg/mL to 80 μg/mL. The results showed that the ZnONPs-based herbal mouthwash at the lowest concentration (5 μg/mL) exhibited the same percentage of live nauplii (90%) as the commercial mouthwash. At higher concentrations (80 μg/mL), the ZnONPs-mediated herbal mouthwash demonstrated 80% live nauplii, indicating lower toxicity compared to the commercial mouthwash.

## Discussion

The current work elucidates the potential of green-synthesized lemongrass and mint-mediated ZnONPs-based herbal mouthwash. The agar well diffusion technique was employed to evaluate the antibacterial activity of ZnONPs. Significant sensitivity against *E. faecalis, S. aureus, S. mutans, and C. albicans* was observed in the findings. The brine shrimp lethality test was used to investigate the crucial problem of toxicity. According to the results, the ZnONPs-mediated mouthwash was found to be less toxic compared to the commercial mouthwash, suggesting that it may be appropriate for use. This result implies that the herbal formulation can provide a substitute with fewer adverse reactions that might contribute to overall well-being.

In parallel, previous studies about green synthesis of ZnONPs prepared using *Parthenium hysterophorus* exhibited a maximum zone of inhibition against *Enterobacter aerogenes* (36 mm), while the least activity was seen against *S. aureus* and *Bacillus subtilis*, in agar well diffusion technique. The antibacterial activity of prepared lemongrass and mint-mediated ZnONPs against *Enterococcus faecalis* and *S. aureus* revealed a notable zone of inhibition. The choice of lemongrass and mint in the synthesis process aligns with the growing interest in harnessing the natural properties of plants for therapeutic purposes, as supported by previous studies [17]. The green synthesis of ZnONPs using *Cinnamomum tamala *leaf extract and their antibacterial activity against *S. aureus* has been shown using a broth dilution study [18]. In a similar study, the green synthesis of ZnONPs demonstrated potential activity against *Xanthomonas oryzae*, emphasizing the broad-spectrum antibacterial potential of nanoparticles, with the data indicating dose-dependent behavior [19]. The synthesis of ZnONPs from *Alstonia scholaris* has been established as well, and these photogenic ZnONPs displayed considerable antibacterial efficacy against fungal species and gram-positive and gram-negative bacteria. While there is no fatality rate at higher dosages, this demonstrates that the cytotoxic impact is dose-dependent [20]. Furthermore, the green-synthesized ZnONPs derived from coriander oleoresin showed encouraging results in cytotoxicity tests as they exhibited lower toxicity [21]. The results also revealed that ZnONPs synthesized from *Abies webbiana* are less toxic. As a result, different nanoformulations with lower quantities of these nanoparticles may be generated that are safe, environmentally friendly, and cost-effective [22]. The cytotoxic impact investigated using a brine shrimp mortality assay with coffee bean and xylitol ZnONPs revealed that 80% of live nauplii were present at a concentration of 80 g/mL, correlating with the current study's cytotoxic result of lemongrass and mint-mediated ZnONPs [23]. Using a co-precipitation method, ammonium hydrogen phosphate and calcium chloride were combined with sodium hydroxide to modify the pH to 8, resulting in the synthesis of amorphous calcium phosphate (ACP) nanoparticles. The produced mouthwash's antibacterial properties were comparable to those of the earlier ACP nanoparticles. Thus, it is possible to regard the ACP nanoparticles as having the ability to obstruct stress-bearing caries restorations. When compared to commercial mouthwashes like Oral-B and chlorhexidine, the produced mouthwash has more benefits, including being natural, less toxic, and inexpensive [24].

This study provides a comprehensive assessment of antimicrobial activity and biocompatibility, revealing compelling evidence for the efficacy and safety of this novel approach in combating oral infections through the use of herbal-mediated ZnONPs-based mouthwashes. The diverse range of inhibited oral pathogens, coupled with minimal cytotoxic reactions, supports the potential of these formulations as viable alternatives in oral care practices.

Limitations

One limitation of this study is its in vitro nature, emphasizing the need for in vivo research to validate the observed antimicrobial efficacy.

## Conclusions

The present study indicates that ZnONPs synthesized through green methods using lemongrass and mint herbal formulations exhibit promising antimicrobial activity against oral pathogens. This suggests their potential use as an effective mouth cleanser to reduce bacterial load in the mouth and for rinsing teeth. However, comprehensive research on ZnONPs is crucial due to their unique properties, as some nanoparticles have shown adverse effects on the oral cavity at higher concentrations. While natural lemongrass and mint-mediated ZnONPs-based mouthwash hold potential therapeutic properties, further investigation is necessary to identify optimal clinical applications for treatment. ZnONPs might contribute to gum health, prevent oral diseases, and show potential as antimicrobial agents in future dentistry.
